# The association between the visceral to subcutaneous abdominal fat ratio and the risk of cardiovascular diseases: a systematic review

**DOI:** 10.1186/s12889-024-19358-0

**Published:** 2024-07-09

**Authors:** Hadi Emamat, Ali Jamshidi, Akram Farhadi, Hamid Ghalandari, Mohadeseh Ghasemi, Hadith Tangestani

**Affiliations:** 1 The Persian Gulf Tropical Medicine Research Center, The Persian Gulf Biomedical Sciences Research Institute, Bushehr University of Medical Sciences, Bushehr, Iran; 2 Department of Nutrition, Faculty of Health and Nutrition, Bushehr University of Medical Sciences, Bushehr, Iran; 3 Department of Community Nutrition, Faculty of Nutrition and Food Sciences, Shiraz University of Medical Sciences, Shiraz University of Medical Sciences, Shiraz, Iran; 4 Students Research Committee, Faculty of Medicine, Bushehr University of Medical Sciences, Bushehr, Iran

**Keywords:** Obesity, Abdominal fat, Visceral fat, Subcutaneous fat, Visceral to subcutaneous fat ratio, Cardiovascular diseases

## Abstract

**Background:**

Cardiovascular diseases (CVDs) are the primary cause of mortality globally. The prevalence of obesity is rising worldwide; there seems to be a significant positive association between obesity and CVDs. The distribution of fat in the abdominal area in the form of visceral (VAT) or subcutaneous adipose tissue (SAT) affects the risk of CVDs. The aim of the present study was to conduct a systematic review of the available literature regarding the association between the VAT-to-SAT ratio and CVDs.

**Methods:**

A comprehensive search strategy was used to retrieve all human observational studies indexed in PubMed, Scopus and Google Scholar databases/search engines (from Jan 2000 up to Oct 2023). The VAT-to-SAT or SAT-to-VAT ratio was an independent variable and various cardiovascular diseases, including hypertension, atherosclerosis, coronary heart disease, cerebrovascular disease and heart failure, were considered as outcomes of interest.

**Results:**

Out of 1173 initial studies, 910 papers were screened. Based on the inclusion criteria, 883 papers were excluded. Finally, 27 papers (18 cross-sectional and 9 cohort studies) published between 2010 and 2023 which met the inclusion criteria were reviewed.

**Conclusions:**

The distribution of abdominal fat seems to be associated with the risk of CVDs; the majority of the evidence suggests that a higher abdominal VAT-to-SAT ratio is associated with the development of CVDs. Therefore, this ratio can be used as a prognostic indicator for CVDs.

**Trial registration:**

Not applicable.

## Introduction

Cardiovascular diseases (CVDs) continue to be the primary cause of mortality on a global scale, significantly affecting both the quality of life of the inflicted and healthcare expenses [1]. CVDs have been identified as the cause of an estimated 20.5 million deaths worldwide in the year 2021 [2]. On the other hand, the prevalence of obesity appears to be ever-increasing globally and obesity has been widely recognized as a complex, multifactorial disorder [3]. The World Obesity Atlas 2023 report states that currently, 38% of the world’s population is experiencing either overweight or obesity [4]. There is a significant positive association between obesity and various types of cancer, metabolic syndrome, CVDs (such as coronary disease, acute myocardial infarction, heart failure, cardiac arrhythmias, and sudden cardiac death), and all-cause mortality [3, 5]. Increased inflammation, insulin resistance, abnormal metabolism of lipids, vascular endothelial dysfunction, and increased blood pressure are considered as the most important underlying mechanisms linking obesity with atherosclerosis [6, 7]. Recently, the use of novel approaches in advanced imaging, biomarkers, genetics and artificial intelligence have been proposed as cardiovascular risk stratification strategies rather than just traditional risk factors [8]. Obesity stems from a condition called adiposopathy, which is characterized by anatomical and functional disturbances in adipose tissue caused by a positive caloric balance in genetically and environmentally vulnerable individuals, which can lead to adverse metabolic, endocrine and immune responses [9].

Body mass index (BMI) is a commonly utilized index to detect adiposity; however, it might ignore some important properties of obesity. Individuals who have similar BMI values may exhibit different cardio-metabolic characteristics, which may indicate an association between the risk of CVDs and the distribution of body fat, independent from the total fat mass of the individual [10]. A comprehensive understanding of the distribution of body fat in individuals with obesity will help further clarify their susceptibility to CVDs [11]. The site of fat accumulation seems substantial in determining whether obesity may lead to metabolic complications such as insulin resistance, metabolic syndrome, type 2 diabetes, and CVDs [12, 13].

Two types of abdominal fat deposits, subcutaneous adipose tissue (SAT) and visceral adipose tissue (VAT), have been extensively studied in relation to metabolic diseases [14, 15]. Even though VAT comprises a small proportion of the overall body fat, research has suggested an association between elevated VAT and the risk of an unfavorable metabolic profile, independent of the total body fat [16]. Several studies have indicated the role of VAT in increasing the risk of cardio-metabolic diseases [17, 18]. On the other hand, SAT is generally considered a neutral fat storage area and has even been suggested to protect against type 2 diabetes and coronary artery disease [16, 19–21].

The absolute amount of VAT has been directly measured using advanced imaging methods, such as computed tomography (CT) scans, to explore its relevance in predicting the risk of metabolic disorders [22]. However, given the distinctive properties of SAT in the pathophysiology of metabolic abnormalities from those of VAT, measuring an index that includes both compartments, i.e., the visceral-to-subcutaneous fat (VAT-to-SAT) ratio, might theoretically provide a more comprehensive insight into the individual’s future risk [23]. A number of studies have examined the association between the VAT-to-SAT ratio and cardio-metabolic risk factors [24–27]. Therefore, given the abundance of existing literature and the rise of controversial invasive fat reduction methods that primarily target SAT, the aim of the present study was to conduct a systematic review of the available literature which investigated the association between the abdominal ratio of VAT-to-SAT and the risk of CVDs.

## Materials and methods

The current systematic review was conducted in accordance with the Preferred Reporting Items for Systematic Reviews and Meta-analyses (PRISMA) guidelines [28]. All human observational studies indexed in PubMed, Scopus and Google Scholar databases/search engines (from January 2000 up to October 2023) were obtained using a comprehensive search strategy. Also, the reference list of included studies was reviewed to find more relevant studies. The following keywords were used to search for relevant studies: [“Visceral to subcutaneous” OR “Subcutaneous to visceral” OR “Visceral fat to subcutaneous fat” OR “Visceral: subcutaneous” OR “Subcutaneous: visceral” OR “Visceral/subcutaneous” OR “Subcutaneous/visceral” OR “Visceral-to-subcutaneous” OR “VAT/SAT” OR “VAT to SAT” OR “Visceral Fat Area to Subcutaneous Fat Area” OR “VFA to SFA” OR “VFA/SFA” OR VSR] AND [hypertension OR HT OR “blood pressure” OR BP OR cardiovascular OR coronary OR heart OR CVD OR CHD OR IHD OR myocardial OR ischemic OR stroke OR “cerebral vascular” OR cerebrovascular OR CVA OR cerebrovascular OR “Heart Failure” OR atherosclerosis].

### Study selection

Observational studies of human populations published in English that investigated the association or correlation between VAT-to-SAT ratio and CVDs were included. In the present study, VAT-to-SAT or SAT-to-VAT ratios were independent variables and various CVDs, including hypertension, atherosclerosis, coronary heart disease, cerebrovascular disease, and heart failure were considered as outcomes of interest.

After eliminating the duplicates, two reviewers (H. E. and H.T) independently verified the titles and abstracts of articles to select the potentially relevant studies to be included in the review. The two reviewers were blinded to the authors. Subsequently, the following pre-defined exclusion criteria were used to exclude irrelevant papers: in-vitro or animal studies, interventional studies, review articles, editorials, non-research letters, ecologic studies, case reports or case series, non-English studies, and studies that did not report the exposure or outcome of interest.

Following the exclusion of irrelevant papers, full texts of the remaining articles were meticulously perused to retrieve eligible ones to be included in the review process. Afterward, the following papers were also excluded: studies that separately examined VAT and SAT, and not VAT-to-SAT/SAT-to-VAT ratio; studies that investigated other outcomes rather than CVDs; articles that merely reported the average of VAT-to-SAT ratio in two groups; and other unrelated studies. Eventually, in the event of any disagreement between the reviewers regarding the inclusion/exclusion of relevant studies, a consensus was reached through a discussion.

### Data extraction and synthesis

At this stage, a pre-established data abstraction form was used to retrieve and register the required data. These data included some general information with regard to the publication (including the first author’s name, the location of the study, the main title of the article, the name of the journal, the date of publication, and the study design), age and biological gender of participants, sample size, health status of the participants, the imaging method used, and CVDs-related outcomes.

### Quality assessment of included studies

The quality of the included studies was evaluated using the Newcastle-Ottawa Scale (NOS) assessment tools for cohort studies [29] and the modified version of NOS adapted for cross-sectional studies [30] (Table 1). The NOS assesses studies based on three categories of criteria: selection (S), comparability (C), and outcome (O). A maximum score of nine points (S: 4, C: 2, and O: 3 points) and ten points (S: 5, C: 2, and O: 3 points) is obtainable for cohort and cross-sectional studies, respectively. In the comparability section, one point was awarded if the study was controlled for sex, age, and smoking confounders, and another point for adjusting for any further covariates (at least two).

**Table 1:** Characteristics of the studies in this systematic review

Author (year)	Country	Sex	Age (year)	Study design (follow-up period)	Participants and sample size	Imaging/ assessment method	Cardiovascular related parameter	Findings	NOS score
Ishikawa et al. [2010]	Japan	M/F	M: 62.9 ± 9.2 F: 64.1 ± 8.1	cross-sectional	572 patients with CVD risk factors and under stable antihypertensive treatment	CT	Hypertension	V/S fat was associated with difficult-to-treat hypertension in men (OR: 1.44, *p* = 0.014).	6
Kaess et al. [2012]	USA	M/F	M: 49.5 ± 10.6 F: 51.8 ± 9.7	cross-sectional	3,223 participants from the Framingham Heart Study	CT	Blood pressure	V/S fat was significantly correlates with cardio-metabolic risk factors such as SBP and DBP (*p* < 0.001).	9
Aoqui et al. [2013]	Brazil	M	59 ± 9.2	cross-sectional	65 non-dialyzing CKD patients	CT	CAC score	Higher V/S fat was positively associated with CAC score, independently of confounders (*p* = 0.007).	7
Kamimura et al. [2013]	Brazil	M/F	55.3 ± 11.3	Cohort (24 months)	113 non-dialyzing CKD patients	CT	Cardiovascular events including acute myocardial infarction, angina, arrhythmia, uncontrolled blood pressure, stroke and cardiac failure	Higher V/S fat was associated with higher risk of cardiovascular events (HR: 8.7, *p* = 0.011).	7
Oike et al. [2014]	Japan	M/F	M: 63 ± 12 F: 65 ± 15	cross-sectional	237 participants who underwent an inpatient medical health checkup	CT	Atherosclerosis (IMT)	There are not any significant associations between V/S fat and atherosclerotic changes.	8
Gast et al. [2015]	Netherlands	M/F	45 to 65	cross-sectional	2451 participants with BMI ≥ 27 kg/m^2^	MRI	Atherosclerosis (CIMT)	A high V/S ratio was associated with larger CIMT.	7
Bouchi et al. [2015]	Japan	M/F	65 ± 12	cross-sectional	148 patients with type 2 diabetes	CT	Atherosclerosis (CIMT)	V/S ratio positively correlated with CIMT in both univariate (β = 0.506, *p* < 0.001) and multivariate linear regression tests (β = 0.383, *p* < 0.001).	9
Figueroa et al. [2016]	USA	M/F	55 (45–5)	Cohort (4 years)	415 subjects who underwent PET and CT imaging for oncological evaluation	CT	Cardiovascular events including incident stroke or transient ischemic attack, acute coronary syndrome, revascularization, new onset angina, peripheral arterial disease, heart failure, or CVD death	V/S ratio was associated with CVD events (HR: 3.60 (1.88–6.92), *p* < 0.001).	6
Kunimura et al. [2016]	Japan	M/F	~ 69	Cohort (8 years)	357 consecutive patients with stable CAD	CT	Cardiac events including cardiac death, non-fatal myocardial infarction, and any revascularization, including target lesion revascularization and revascularization of new lesions	The HR of high V/S for CVD events was 2.72 (1.04–7.09, *p* = 0.04) compared with the low V/S.	8
Ladeiras-Lopes et al. [2017]	Portugal	M/F	57.7 ± 10.2	Cohort (1.3 years)	713 participants without known heart disease	CT	Cardiac events including myocardial infarction or a revascularization procedure and death	The V/S ratio was an independent predictor of death and cardiac events (HR: 1.43 (1.03–1.99)).	7
Higuchi et al. [2017]	Japan	M/F	59.0 ± 11.5	cross-sectional	3007 apparently healthy adults	CT	Small and large cerebrovascular lesions including ischemic change, cerebral artery stenosis or occlusion and cervical plaque	The V/S ratio was independently related to small and large cerebrovascular lesions (OR = 1.05, 1.12 and 1.09 for ischemic change, cerebral artery stenosis or occlusion and cervical plaque respectively, *p* < 0.05).	9
Yun Hwan Oh et al. [2017]	South Korea	M/F	M: 52.1 ± 9.9 F: 50.6 ± 9.7	cross-sectional	535 subjects with normal WC	CT	Hypertension	V/S ratio appeared to be an independent predictor of the multiple metabolic risk factors such as hypertension in both men and women (*P* < 0.001). In men, V/S ratio was superior to VFA (*P* = 0.028).	6
Glesby et al. [2018]	USA	F	42	cross-sectional	244 women with and 99 without HIV infection	DXA	Atherosclerosis (carotid artery stiffness, presence of carotid artery lesions, and CIMT)	The V/S ratio was not statistically associated with any of the outcomes after confounder’s adjustment.	8
Yoshida et al. [2018]	Japan	M/F	61.8 (± 11.9)	cross-sectional	352 consecutive outpatients with rheumatoid arthritis	BIA	Atherosclerosis (IMT and Plaque score (PS) of the carotid artery)	The V/S ratio was independently associated with the IMT (*p* = 0.037) and PS (*p* = 0.043).	9
Fukuda et al. [2018]	Japan	M/F	64 ± 13	retrospective cohort (2.5 years)	682 patients with type 2 diabetes	dual BIA	CVD including stroke, unstable angina, myocardial infarction, percutaneous coronary intervention, coronary bypass grafting, angioplasty or major amputation as a result of peripheral arterial disease or cardiovascular death	The V/S ratio was positively associated with incident or recurrent CVD (HR = 1.82, 95% CI: 1.09–3.04, *P* = 0.021).	7
Gao et al. [2018]	China	M/F	63.90 ± 11.96	cross-sectional	223 In-patients	non-enhanced CT	CAD	The V/S ratio was significantly associated with CAD (OR = 10.49, *p* < 0.001)	8
Ju-Yeon Yu et al. [2019]	South Korea	M/F	37.4 ± 12.7	cross-sectional	145 men and 455 women who received health checkups	CT	Hypertension	V/S was significantly related to DBP (p for trend < 0.05).	7
Abe et al. [2019]	Japan	M/F	10–15	retrospective cohort	61 adolescents	CT	Blood pressure	V/S ratio was not correlated with SBP and DBP.	4
Miura et al. [2019]	Japan	M/F	73 ± 13	retrospective cohort (3 years)	111 patients diagnosed with acute aortic dissection	CT	Major Adverse Cardiovascular and Cerebrovascular Events (MACCE) including all-cause mortality, acute myocardial infarction, recurrent aortic dissection, aorticenlargement, or acute ischemic stroke	The V/S ratio tends to be associated with the 3-year MACCE (HR = 1.49, *p* = 0.05).	6
Okada et al. [2020]	Japan	M/F	64 ± 12	retrospective cohort	60 patients with acute coronary syndrome	CT	Coronary plaque instability	Higher V/S ratio was independently associated with higher coronary plaque vulnerability (*p* = 0.03).	6
Bogorodskaya et al. [2020]	USA	M/F	M: 47 ± 7 F: 46 ± 7	cross-sectional	148 people with HIV and 68 uninfected individuals without CVD	CT	Atherosclerotic coronary plaque	The V/S ratio showed a strong association with presence of plaque (OR = 3.30, *p* = 0.03) and CAC > 0 (OR = 3.57, *p* < 0.001).	8
Chiyanika et al. [2021]	China	M/F	48 ± 10	cross-sectional	625 participants	MRI	Hypertension	V/S ratio significantly correlated with hypertension (*r* = 0.255, *p* < 0.001).	8
Otagiri et al. [2021]	Japan	M/F	66.7 ± 13.0	cross-sectional	942 suspected CAD patients	CT	CAD	The V/S ratio was independent predictor for CAD severity (β = 0.25; *p* < 0.001).	9
Pereira-Manfro et al. [2021]	Brazil	M/F	49	cross-sectional	309 Brazilian civil servants	DEXA	Cardiovascular risk-score	In both sexes, V/S ratio were directly associated with CV risk-score (*p* < 0.05).	9
Wibmer et al. [2022]	USA	M/F	31 (15–58)	Cohort (26 months)	455 patients	CT	Framingham-estimated risk of CVD	V/S significantly associated with Framingham-estimated risk of CVD (OR = 1.43 (1.21 to 1.69), *p* < 0.001).	5
Wang et al. [2022]	China	M/F	67.19 ± 8.68	cross-sectional	186 patients	CT	Intracranial atherosclerotic stenosis (ICAS)	The V/S ratio was independent predictor of ICAS (OR = 26.08 (5.92–114.83); *p* < 0.001).	9
Liu et al. [2023]	USA	M/F	41.35 ± 11.16	cross-sectional	4,899 obese participants	DEXA	CCVD (such as hypertension, coronary heart disease, heart failure, and stroke)	The V/S ratio was significantly related to the CCVD comorbidities (*p* < 0.05).	9

*Abbreviation* CVD: Cardiovascular Diseases; CT: Computed Tomography; V/S: visceral-to-subcutaneous fat ratio; CKD: Chronic Kidney Disease; CAC: coronary artery calcium; HR: Hazard Ratio; IMT: intima-media thickness; BMI: Body Mass Index; BIA: bioelectrical impedance analysis; CIMT: carotid intima-media thickness; PET: positron emission tomography; OR: Odds Ratio; WC: Waist Circumference; VFA: Visceral Fat Area; HIV: Human Immunodeficiency Virus; PS: Plaque Score; CAD: Coronary Artery Disease; DBP: Diastolic Blood Pressure; SBP: Systolic Blood Pressure; MACCE: Major Adverse Cardiovascular and Cerebrovascular Events; MRI: Magnetic Resonance Imaging; DEXA: Dual-Energy X-ray Absorptiometry; CV: Cardio Vascular; ICAS: Intracranial Atherosclerotic Stenosis; CCVD: Cardio-Cerebrovascular Diseases

## Results

### Study selection

**Fig. 1:**
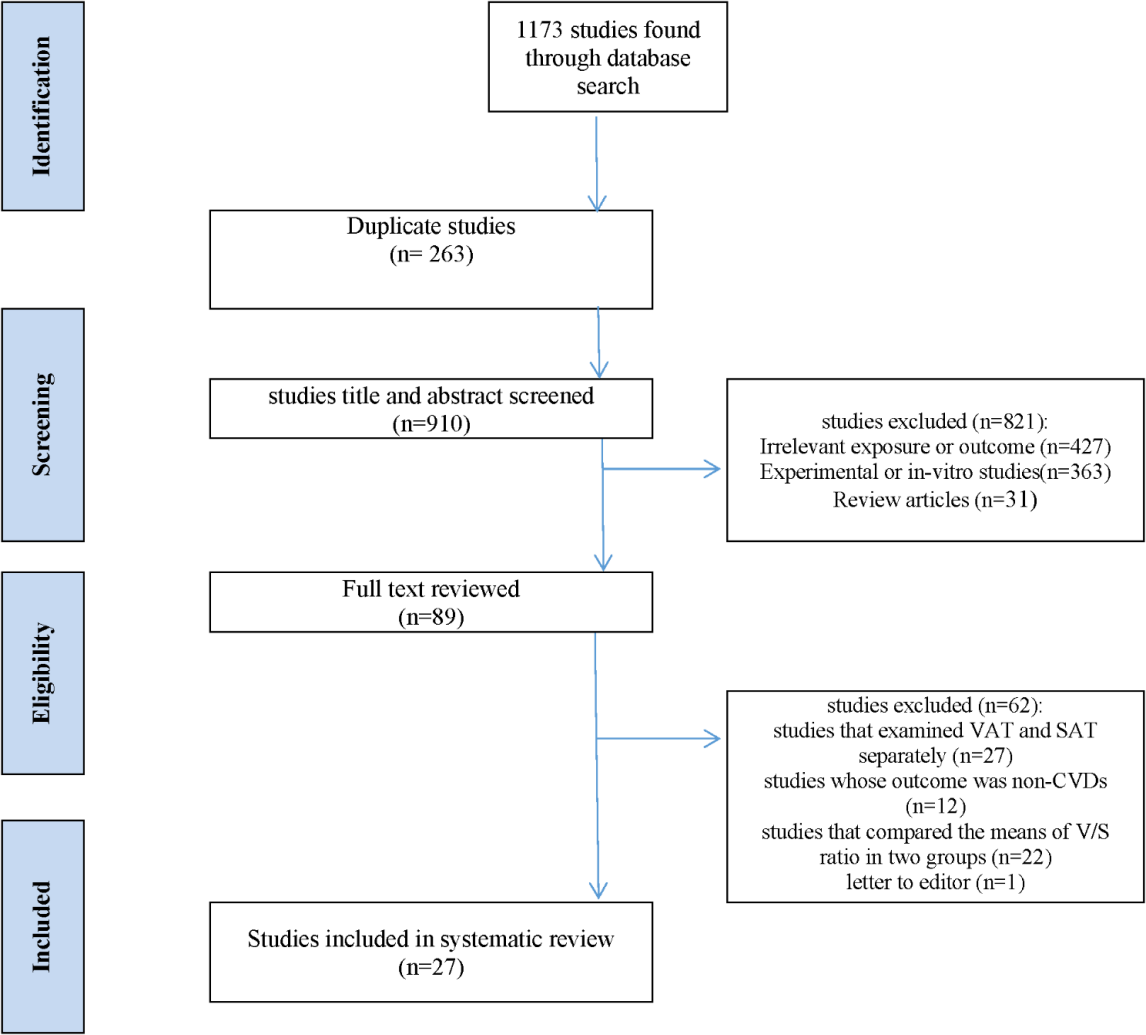
PRISMA flow diagram of review process

The study selection process is presented in Fig. 1. Out of 1173 studies initially retrieved, 263 were duplicates and excluded. Afterwards, the remaining 910 papers were screened. A total of 883 papers that did not meet the inclusion criteria were excluded. Finally, 27 papers (18 cross-sectional and 9 cohort studies) published between 2010 and 2023, which met our inclusion criteria, were included. Amongst the included studies, six articles examined the association between VAT-to-SAT ratio and blood pressure (BP) [15, 23, 31–34], 9 studies examined atherosclerosis indices [24, 35–42], 10 studies examined CVDs events [26, 43–51] and remaining two studies assessed CVDs risk scores [25, 52]. Among the reviewed studies, eleven were conducted in Japan [26, 31, 33, 36, 38, 39, 41, 43, 47, 48, 51], six in USA [15, 40, 42, 44, 49, 52], three in Brazil [25, 35, 45], three in China [24, 34, 50], two in South Korea [23, 32], one in Netherlands [37], and one in Portugal [46]. The characteristics and quality scores of the included studies are shown in Table 1.

### Main results

#### VAT-to-SAT ratio and blood pressure (BP)

The association between BP and VAT-to-SAT ratio has been investigated in six research studies. In a cross-sectional investigation conducted by Kaess et al. [15], which involved 3,223 participants from the Framingham Heart Study, a significant correlation was observed between the VAT-to-SAT ratio and cardio-metabolic risk factors. They reported a significant association between the VAT-to-SAT ratio and systolic and diastolic BP (*p* < 0.001). Yun Hwan Oh et al. [23] conducted a cross-sectional study on 535 Korean individuals with a normal waist circumference. They observed that the VAT-to-SAT ratio is an independent predictor for multiple metabolic risk factors, including hypertension (HTN) in both sexes (*p* < 0.001). Additionally, their findings suggest that the VAT-to-SAT ratio is a better predictor of HTN than visceral fat area (VFA) (*p* = 0.028). Chiyanika et al. [34] observed a correlation (*r* = 0.255) between HTN and VAT-to-SAT ratio (*p* < 0.001) in a population of 625 Chinese. In a cross-sectional study conducted by Ishikawa et al. [31] in 2010, which involved 572 Japanese participants with cardiovascular risk factors and stable antihypertensive treatment, a significant association was observed between the VAT-to-SAT ratio and difficult-to-treat hypertension in male subjects (OR: 1.44, *p* = 0.014). However, in a cross-sectional study of 600 subjects, Ju-Yeon Yu et al. [32] reported a significant association between the VAT-to-SAT ratio and only diastolic BP (p for trend < 0.05). In contrast, Abe et al. [33] reported that there was no association between VAT-to-SAT ratio and neither systolic nor diastolic BP in 61 Japanese adolescents aged 10 to 15 years old.

#### VAT-to-SAT ratio and atherosclerosis

Gast et al. [37] and Bouchi et al. [38] both reported a significant association between a higher VAT-to-SAT ratio and elevated carotid intima-media thickness (CIMT), in cross-sectional studies on individuals inflicted with overweight (BMI ≥ 27 kg/m^2^) and type 2 diabetes; respectively. Moreover, Yoshida et al. [41] observed a significant association between VAT-to-SAT ratio and IMT (*p* = 0.037) and plaque score (*p* = 0.043) in 352 Japanese patients with rheumatoid arthritis. Aoqui et al. [35], in a cross-sectional study on Brazilian male subjects with non-dialyzing chronic kidney disease (CKD) aged 59 ± 9.2, indicated that a higher VAT-to-SAT ratio was independently associated with the coronary artery calcium (CAC) score (*p* = 0.007). Moreover, Bogorodskaya et al. [42] suggested that there is a correlation between VAT-to-SAT ratio and CAC score > 0 (OR = 3.57, *p* < 0.001) in CVD-free patients with human immunodeficiency virus (HIV) as well as an increased risk of the presence of atherosclerotic plaques (OR = 3.30, *p* = 0.03). According to the study conducted by Wang et al. [24], the VAT-to-SAT ratio was proposed as an independent predictor for intracranial atherosclerosis (ICAS) (OR = 26.08; 95% confidence interval (CI): 5.92–114.83; *p* < 0.001) in a sample of 186 patients with an average age of 67.19 ± 8.68. Higuchi et al. [39] conducted a cross-sectional study on 3007 healthy Japanese adults. They observed that the VAT-to-SAT ratio was independently and significantly associated with the presence of small and large cerebrovascular lesions (OR = 1.05, 1.12, and 1.09 for ischemic change, cerebral artery stenosis or occlusion, and cervical plaque; respectively, *p* < 0.05). However, in a study involving 237 individuals who underwent an inpatient medical health check-up, Oike et al. [36] found that there was no significant correlation between atherosclerotic changes and the VAT-to-SAT ratio. Furthermore, in a cross-sectional study examining 244 women with HIV and 99 without HIV in the United States, Glesby et al. [40] reported no statistical association between the VAT-to-SAT ratio, and any of the atherosclerotic markers (including carotid artery stiffness, presence of carotid artery lesions, and CIMT) after adjusting for confounders.

#### VAT-to-SAT ratio and cardiovascular events

Seven studies that examined the association between the VAT-to-SAT ratio and the incidence of cardiovascular events were included in the present investigation. Kamimura et al. [45] conducted a study on 113 non-dialyzing patients with CKD, Figueroa et al. [44] studied 415 subjects who underwent positron emission tomography (PET) and CT imaging for oncological evaluation, and Kunimura et al. [43] studied 357 patients with stable coronary artery disease. All three cohort studies found a strong correlation between the VAT-to-SAT ratio and an elevated risk of cardiovascular events, with hazard ratios (HRs) of 8.7, 3.6, and 2.72, respectively. Similarly, Ladeiras-Lopes et al. [46] reported that the VAT-to-SAT ratio was an independent predictor for death and cardiac events (HR = 1.43; 95%CI, 1.03–1.99) in a cohort study of 713 individuals without known heart disease in Portugal. Fukuda et al. [47] conducted a study on 682 individuals with type 2 diabetes; they observed that the VAT-to-SAT ratio had a positive correlation with the occurrence or recurrence of CVDs (HR = 1.82, 95% CI: 1.09–3.04, *P* = 0.021). In a retrospective cohort study on 60 patients with acute coronary syndrome, Okada et al. [26] reported that a higher VAT-to-SAT ratio was positively correlated with an augmented susceptibility to coronary plaque (*p* = 0.03). Furthermore, Miura et al. [48] reported a significant association between the VAT-to-SAT ratio and major adverse cardiac and cerebrovascular events (MACCEs), including all-cause mortality, acute myocardial infarction, recurrent aortic dissection, aortic enlargement, or acute ischemic stroke (HR = 1.49, *p* = 0.05) in 111 patients diagnosed with acute aortic dissection. Another study by Liu et al. [49], conducted on 4899 individuals with obesity, revealed that the VAT-to-SAT ratio was significantly related to cardiovascular and cerebrovascular comorbidities (such as hypertension, coronary heart disease, heart failure, and stroke) (*p* < 0.05). Two studies by Gao et al. [50] and Otagiri et al. [51] investigated the association between a higher VAT-to-SAT ratio and CAD. The first one, which involved 223 inpatients from China, reported a significant association (OR = 10.49, *p* < 0.001). The latter, conducted on 942 individuals in a cross-sectional design, reported the VAT-to-SAT ratio to be an independent predictor for CAD severity (β = 0.25; *p* < 0.001).

#### VAT-to-SAT ratio and cardiovascular Risk scores

Wibmer et al. [52], in a study on 455 individuals, reported a significant association between the VAT-to-SAT ratio and Framingham-estimated risk of CVDs within ten years (OR = 1.43; *p* < 0.001). In addition, Pereira-Manfro et al. [25], in investigating 309 Brazilian civil servants, observed that the VAT-to-SAT ratio was associated with a CV risk score developed by the ACC/AHA [53] (*p* < 0.05) in both biological genders. There are no results referring to other validated risk scores.

## Discussion

The present systematic review sums up the existing literature in the form of observational studies regarding the association between the abdominal VAT-to-SAT ratio and the risk of CVDs. The pooled evidence suggests a positive association between the abdominal VAT-to-SAT ratio and the risk of various CVD-related outcomes. Overall, only in three out of 27 included studies did the association diminish following the adjustment of the confounders. Therefore, the abdominal VAT-to-SAT ratio seems to be relevant in predicting the risk of CVDs.

Abdominal obesity has been historically suggested as a risk factor for CVDs [54, 55]. However, according to the obesity paradox, in certain obese subpopulations, the mortality rate from CVD is lower, and this observation can highlight the fundamental role of fat distribution in the body [56]. The underlying mechanisms leading to individual differences in body fat distribution are multifaceted and need to be fully understood yet. However, research suggests that factors such as genetics, sex hormones, the use of medications (e.g., glucocorticoids), and epigenetics all contribute to how excess calories from the diet are stored within the abdominal region [57].

Abdominal adiposity could be divided into two main compartments, namely SAT and VAT [58]. SAT and VAT differ in various aspects, such as anatomical structure, cellular composition, molecular makeup, physiological functions, clinical implications, and prognostic significance [58]. The available evidence suggests a consensus regarding the effects of fat accumulation in the form of VAT in increasing an individual’s vulnerability to cardio-metabolic risk [59, 60], but the evidence regarding SAT is rather contradictory. Despite the fact that many studies indicate that body fat mass and, in parallel, SAT are related to the occurrence of CVDs [61, 62], some evidence suggests that SAT might not be the main culprit as previously assumed. For instance, it has been proposed that the accumulation of fat in the form of SAT (rather than VAT) might have a protective impact against atherosclerosis in asymptomatic patients [21]. Furthermore, SAT has been hypothesized to be negatively associated with the risk of insulin resistance [63] and diabetes [19].

It has been proposed that the reduction of SAT expansion and subsequent increase in its density might be the leading factor in increasing ectopic fat accumulation, i.e., VAT. Several mechanisms have been suggested as interdependent mechanisms in reducing SAT storage capacity, including reduction of angiogenesis, up-regulation of inflammatory pathways, fibrosis of the adipose tissue, physical remodeling of adipocytes, and changes in cellular lipid trafficking [64]. According to the latter hypothesis, SAT has been suggested to function as a protective metabolic reservoir, and when it reaches its maximum capacity, any excess energy is stored as VAT [11]. Thus, it might be safe to assume that the VAT-to-SAT ratio can outperform VAT alone in predicting cardio-metabolic risk [23], confirming the present study’s findings.

Not all of the included studies reported the abdominal VAT-to-SAT ratio as a risk factor for CVDs. For instance, Oike et al. [36] failed to observe any associations between the VAT-to-SAT ratio and changes in IMT. However, it seems that selection bias could have played a role; the researchers enrolled participants from a single medical check-up center, which resulted in an imbalance in the final study population by selecting mainly male subjects. However, other studies with larger sample sizes that enrolled overweight or diabetic participants tend to approve of the existence of such an association [37, 38]. Glesby et al. observed that although VAT (positively) and SAT (negatively) are associated with the risk of arterial disorders, the VAT-to-SAT ratio does not seem relevant. Likewise, such discordance appears to have originated from their selection process, which enrolled female subjects with HIV who, by the effects of the disease and the potent antiviral medications, might show significant differences in body composition and fat metabolism, specifically in their fat deposits [40]. Another study observed no association between VAT-to-SAT ratio and blood pressure [33]. Following the same pattern, involving a very small sample size (61 subjects) with specific properties (adolescents who have undergone multiple drastic metabolic and physiological changes) might have nullified the potential association otherwise observed by higher-quality investigations [15, 23, 31].

There exist some justifications for how histological and metabolic distinctions between VAT and SAT might differentiate their effects on risk factors of CVDs [58, 65]. Anatomically, VAT is mainly found in the mesentery and omentum. The venous blood of VAT flows directly to the liver through the portal vein due to its location. In contrast, SAT is drained through systemic veins [66]. Compared to SAT, VAT mainly secretes pro-inflammatory cytokines (including interleukin (IL)-12p70, IL-13, tumor necrosis factor- α (TNF-α), IL-6, and IL-8), adipokines, and prostanoids [16]. The portal drainage of VAT provides the liver with direct access to free fatty acids (FFAs) and adipokines [58]. Subsequently, adipokines stimulate hepatic immune mechanisms and result in the production of several inflammatory mediators [15, 58]. Furthermore, adipocytes in the VAT exhibit higher metabolic activity, are more susceptible to the breakdown of fats, and have a greater resistance to insulin (owing to the lower expression of insulin receptor substrate-1(IRS-1)) in comparison to SAT [67]. Moreover, VAT displays greater potential for producing FFAs and absorbing glucose, is more responsive to the stimulation from the nervous system; on the contrary, SAT is more efficient in absorbing FFAs and triglycerides which are circulating in the bloodstream [58].

The main limitation of this systematic review is that most of the included studies have cross-sectional and case-control designs, which makes deriving a causal inference impossible. Based on that, further prospective observational studies are needed to confirm the deductions made in the present study. However, to the best of our knowledge, the current study is the first attempt to systematically investigate the existing literature regarding the possible association between the VAT-to-SAT ratio and the risk of CVDs. The present study’s findings raise an important question regarding the clinical effectiveness of invasive fat-reducing procedures that tend to aim prominently at removing SAT [68], at least when it is conducted to reduce the individual’s risk of infliction with CVDs. Nevertheless, owing to the observational nature of the present study, it seems rational that such a hypothesis be examined through meticulously designed, high-quality clinical trials on individuals with various health statuses. Moreover, in clinical settings, it is still prudent to follow the existing lifestyle-modifying protocols with the aim of reducing the risk of CVDs.

## Conclusion

The findings of the present investigation suggest that a higher abdominal VAT-to-SAT ratio might be associated with the development of CVDs. Therefore, the ratio is relevant as a prognostic indicator for CVDs, and to reduce the burden of CVDs, it is recommended that lifestyle modification strategies that reduce VAT be focused on instead of using methods that only eliminate SAT in clinical practice.

## Data Availability

The tables in this published article contain all the data generated or analyzed during this study.
